# Effects of Alcohol Extracts From *Ganoderma resinaceum* on Sleep in Mice Using Combined Transcriptome and Metabolome Analysis

**DOI:** 10.3389/fnut.2022.745624

**Published:** 2022-01-28

**Authors:** Tianci Chen, Fangyi Zhang, Juanqin Chen, Qiangui Zhong, Yuxin Hu, Ruru Wu, Baogui Xie, Yuji Jiang, Bingzhi Chen

**Affiliations:** ^1^College of Food Science, Fujian Agriculture and Forestry University, Fuzhou, China; ^2^Mycological Research Center, Fujian Agriculture and Forestry University, Fuzhou, China; ^3^Yongtai Agricultural and Rural Bureau, Fuzhou, China

**Keywords:** *Ganoderma resinaceum*, alcohol extract, sleep, metabolome, transcriptome

## Abstract

*Ganoderma resinaceum* is a valuable Chinese medicine. This study aimed to investigate whether a *G. resinaceum* alcohol extract (GRAE) improves sleep, and analyze the potential mechanism. After 30 days of continuous administration of GRAE at various doses, GRAE (1,000 mg/kg.bw) prolonged pentobarbital sodium-induced sleep, increased the rate of sleeping in mice treated with a subthreshold dose of pentobarbital sodium, and shortened sleep latency. The mice brain was analyzed using UPLC-MS/MS and RNA-sequencing. Metabolomics analysis revealed that 73 metabolites in the high-dose (HD) group had changed significantly, mainly in amino acids and their derivatives, especially the accumulation of L-glutamine and PGJ2 (11-oxo-15S-hydroxy-prosta-5Z, 9, 13E-trien-1-oic acid). Transcriptome analysis revealed 500 differential genes between HD and control groups, mainly enriched in neuroactive ligand-receptor interaction, amphetamine addiction, and cocaine addiction pathways. The conjoint analysis of the transcriptome and metabolome showed that the biosynthesis of L-glutamine might be regulated by *Homer1, Homer3*, and *Grin3b*. This suggests that GRAE may affect L-glutamine accumulation by regulating the expression of these genes. This study showed that GRAE may prolong the sleep time of mice by reducing the accumulation of L-glutamine and deepens our understanding of the regulatory network between certain genes and L-glutamine.

## Introduction

Sleep is an important process in the maintenance of health (1). Chronic insomnia may cause regulatory imbalances, leading to various complications (2, 3). It is estimated that over one-third of people suffer from insomnia, and the frequency is increasing (4). At present, most sleep-inducing medicines are synthetic and cause major side effects, such as muscle relaxation, memory loss, and drug dependence (5). Benzodiazepines, the primary drugs used to treat sleep disorders, mainly act on the central nervous system, and long-term use has side effects such as tolerance and dependency (6). Therefore, a safe and efficient alternative is needed to improve sleep quality.

*Ganoderma lucidum* contains polysaccharides, proteins, amino acids, fatty acids, terpenes, steroids, alkaloids, and phenolic compounds. Among these, the water-soluble polysaccharides and alcohol-soluble triterpenoids appear to the main bioactive components (7, 8). *Ganoderma lucidum* is widely used to lower blood pressure, to protect the liver, and as an anti-aging agent in China. Modern pharmacology indicates that *G. lucidum* can improve immune regulation and has antiviral, anti-inflammatory, antioxidant, and anti-tumor effects (9, 10). Many Chinese medical doctors reported that *G. lucidum* can improve sleep in patients with neurasthenia and mental confusion. Previous studies have shown that *G. lucidum* can reduce spontaneous motor activity, prolong non-rapid eye movement (NREM), and relax the central nervous system (11, 12). *Ganoderma lucidum* can improve sleep by increasing the number of GABAA receptors (13). It increases interleukin (IL)-1β, tumor necrosis factor (TNF)-α, and nitric oxide (NO) production in a concentration-dependent manner (14). TNF-α is a key factor in regulating human sleep. Although previous studies have indicated that *G. lucidum* can affect sleep, there is no direct evidence that it can improve sleep.

We previously isolated and identified three strains of *G. resinaceum* from different areas of *G. lucidum* production in China and found that the triterpene content in the fruiting body is higher in *G. resinaceum* than in *G. lucidum* (15). *Ganoderma resinaceum* has been used to prevent and treat diseases such as inflammation and cancer; however, studies of the species are limited. Previous studies have mostly focused on the compositions and activities of *G. resinaceum* polysaccharides and triterpenoids (16, 17). Since it is not clear whether *G. resinaceum* improves sleep, one aim of the current study was to evaluate the *in vivo* effects of *G. resinaceum* on sleep. A second aim of the study was to elucidate the potential effects of *G. resinaceum* alcohol extract (GRAE) on the regulatory networks between genes and key metabolites using transcriptomic and metabolomic analyses.

## Materials and Methods

### Drugs and Drug Administration

*Ganoderma resinaceum* was provided by the Mycological Research Center, Fujian Agriculture and Forestry University. The method for preparing *G. resinaceum* alcohol extract (GRAE) was adopted from a previous study, with minor modifications (18). The fruiting bodies of *G. resinaceum* were dried at 70°C for 12 h, crushed using an herbal grinder, and passed through a 60-mesh sieve. To obtain a solid-liquid ratio of 1:30, ethanol (55% v/v) was added. After passing through a water bath at 60°C for 2 h and filtering under reduced pressure, the first filtrate and the first filter residue were obtained. These two steps were repeated twice to obtain the respective filtrates. The three filtrates were combined and added to a rotary evaporator and rotary evaporated at 55°C. After observing that no significant amount of liquid spiraled out, the temperature was slowly increased to 95°C, during which the extract was kept boiling, and the concentrate was collected and then dried in a freeze drier for 24 h.

### Animals

Male ICR mice (19–21 g) were provided by Shanghai SLAC Laboratory Animal Co. Ltd. (Shanghai, China). Mice were housed in cages with controlled ambient temperature (24 ± 1°C), relative humidity (60 ± 10%), and a 12 h light-dark cycle. Before the experiment, the mice were reared for 1 week to adapt to the environment. In this study, all animal experiments were performed in strict accordance with the European Community guidelines for the use of experimental animals and the rules of the guide for the care and use of laboratory animals published by the US National Institutes of Health (NIH Publication no. 85-23, revised 1996). The animal protocol was approved by the Animal Care and Use Committee of the College of Food Science, Fujian University of Agriculture and Forestry (Protocol code number FS-2019-0055, approved on October 10, 2019).

Wang et al. demonstrated that the ethanol extract of fruiting bodies of *G. lucidum* has no acute and genetic toxicity test in mice using the Ames test, micronucleus test of bone marrow cell, and sperm shape abnormality test (19). In this study, we determined that GRAE at 100-1,000 mg/kg.bw had different degrees of sleep improving effects through preliminary experiments, and selected gradient doses of 250, 500, and 1,000 mg/kg.bw for further experimentation.

GRAE was administered to mice by gavage according to the body weight of mice. ICR mice, with a body weight difference of 2 g, were randomly divided into four groups: control (CK, distilled water), low-dose (LD, 250 mg/kg.bw), middle-dose group (MD, 500 mg/kg.bw), and high-dose (HD, 1,000 mg/kg.bw) groups. The treatments were administered every morning, from 9:00 to 10:00 am, *via* gavage for 30 days. The dosage of each drug was 0.2 ml/10 g.bw.

### Sleep Improvement Experiment

The GRAE improved sleep experiment was conducted according to the method in the “Technical Standards for Testing and Assessment of Health Food (2003 edition) prepared by the Ministry of Health of the People's Republic of China” and the method of Chu et al. (12). Sleep was defined as the disappearance of righting reflex, in which mice remained in a dorsal posture for 30–60 s. Animal wakening was identified as the disappearance of the supine position and limbs touching the ground. A quiet sleep experiment environment was maintained at a temperature of 24°C and a relative humidity of 60 ± 10%. Sleep improvement was evaluated by a previously described method, with some modifications (20). The concentrations of pentobarbital sodium and barbiturate sodium in this experiment were the lowest concentrations that could cause sleep in mice, as verified by preliminary experiments.

#### Induction of Prolonged Sleep by Pentobarbital Sodium

Twenty minutes after the last gavage, the mice were injected intraperitoneally with pentobarbital sodium (55 mg/kg) at a dose volume of 0.2 ml/20 g.bw. The sleep duration of mice was recorded by the righting reflex, from disappearance to recovery. The time of sleep was compared between the treatment and control groups.

#### Application of Pentobarbital Sodium at a Subthreshold Dose

Twenty minutes after the last gavage, the mice were injected intraperitoneally with pentobarbital sodium (32 mg/kg) at a dose volume of 0.2 ml/20 g.bw. The disappearance of the righting reflex indicated the mouse fell asleep. The frequency of falling asleep was compared between mice in the treatment group and the control group.

#### Time Until Barbital Sodium-Induced Sleep

Twenty minutes after the last gavage, the mice were injected intraperitoneally with barbital sodium (280 mg/kg) at a dose volume of 0.2 ml/20 g.bw. The time from administration to the disappearance of the righting reflex was recorded and compared with the control group.

### Mouse Brain Metabolism

After 30 days of continuous administration of GRAE, mice were euthanized and dissected. Mouse brain tissues were frozen immediately with liquid nitrogen and stored at −80°C for later use. The samples were thawed on ice and 50 mg of the sample was supplemented with 1,000 μl of pre-cooled extractant (70% methanol aqueous solution containing 1 μg/ml 2-chlorophenylalanine as an internal standard) and pre-cooled steel balls. The mixture was homogenized for 30 min at 30 Hz and vortexed for 1 min. After standing on ice for 15 min, samples were centrifuged at 10,000 ×g for 10 min. The supernatant was obtained for ultra-performance liquid chromatography tandem mass spectrometry (UPLC-MS/MS) using the Shim-pack UFLC (SHIMADZU CBM30A; Kyoto, Japan) equipped with the Waters ACQUITY HSS T3 C18 (1.8 μm, 2.1 × 100 mm). Phase A was ultrapure water (0.04% acetic acid) and phase B was acetonitrile (0.04% acetic acid). The flow rate was 0.4 ml/min, the column temperature was 40°C, and the injection volume was 2 μl. The elution gradient was: 0 min water/acetonitrile (95: 5 V/V), 11.0 min (5: 95 V/V), 12.0 min (5: 95 V/V), 12.1 min (95: 5 V/V), and 14.0 min (95: 5 V/V).

For tandem mass spectrometry (MS/MS, QTRAP®), the temperature for electrospray ionization was 500°C and the mass spectrometer voltage was 5,500 V for the positive pole and −4,500 V for the negative pole. The other parameters were an ion source gas I of 55 psi, a gas II of 60 psi, and a curtain gas of 25 psi. The collision-activated dissociation parameter was high. Metabolite quantification was accomplished by a multiple reaction monitoring (MRM) analysis with triple quadrupole mass spectrometry. Based on the target standard database MWDB (metware database), qualitative analysis is performed according to the retention time, ion pair information and secondary spectral data of the detected substances (21).

Raw data were converted to mzXML format by ProteoWizard, then peak alignment, retention time correction and extraction of peak areas were performed using the XCMS program. Metabolite structure identification was performed by exact mass number matching (<25 parts per million) and secondary spectrum matching, using the company's own database for searching. Differential metabolites discriminant analysis was performed using OPLS-DA in R studio software (3.2-ZOL). Differential metabolism was selected according to fold change ≧ 2, fold change ≦ 0.5 and VIP (variable importance for the projection) ≧ 1.

### Transcriptomics Analysis of Mice Brain Tissue

The RNA-sequencing (RNA-Seq) experimental method of mice brain tissue was referenced from Wang et al. (22). Three biological replicates were performed for each group. After the final transcriptomic data was generated, stringTie and edgeR were used to estimate the expression levels of all transcripts. StringTie was used to perform expression level for mRNAs by calculating fragments per kilobase per million (FPKM). The differentially expressed mRNAs and genes were selected with log2 (fold change) >1 or log2 (fold change) < -1 and with statistical significance (*P* < 0.05, multiple test corrected *p*-value) using R package (3.2.5). Pathways were built using Kyoto Encyclopedia of Genes and Genomes (KEGG) and OmicShare tools for enrichment analyses.

### Conjoint Analysis of Transcriptome and Metabolome

Conjoint analysis of transcriptome and metabolome were used to comprehensively analyze the effects of GRAE on prolonging sleep time in mice. All differentially expressed genes and metabolites were queried and mapped to pathways based on KEGG. Key metabolic pathways were screened and calculation of Pearson correlation coefficients for genes and metabolites using the cor program in R were used to analyze gene and metabolite correlations. Pathway enrichment analysis was performed based on *p*-values. The correlation between differential genes and metabolites was analyzed using R software, and heatmaps were plotted using the clustermap function.

### Statistical Analysis

Experimental data were analyzed using SPSS 16.0 and R packages (3.2.5), and plots were generated using GraphPad Prism 6 and R packages (3.2.5). All values are presented as the means ± standard deviation (SD). Correction of *p*-values in transcriptome and metabolome was performed using false positive rate (FDR) error control method. Each group of experiments was designed in three parallel replicates, and the experimental results conforming to a normal distribution were analyzed using one-way analysis of variance (ANOVA) followed by Student-Newman-Keuls test. Differences with *P* < 0.05 were considered significant.

## Results

### Effect of GRAE on Sleep Time

**Figure 1:**
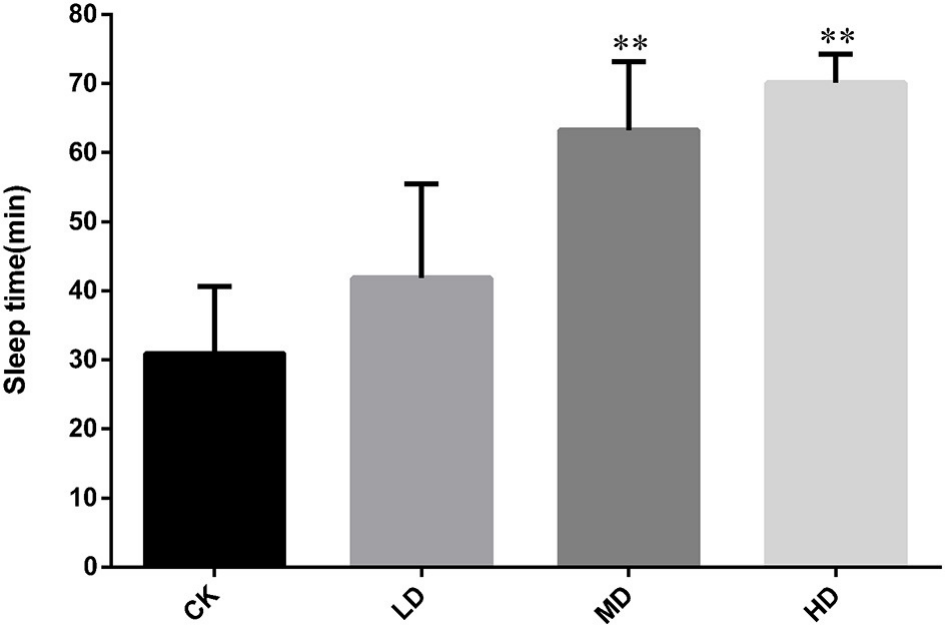
Effect of GRAE on the sleep time of mice induced by pentobarbital sodium in sleep improvement experiment. Each bar represents the mean ± SD (*n* = 10). *P < 0.05 vs. control group, **P < 0.01 vs. control group.

As shown in Figure 1, the length of sleep induced by pentobarbital sodium (55 mg/kg) did not differ significantly between mice in the LD and CK groups (*P* > 0.05). Compared with the sleep duration in CK, the durations in the MD and HD groups were significantly different (*P* < 0.01). The sleep durations of the MD and HD groups were 2.04 and 2.30 times longer than those in CK, respectively.

In mice treated with pentobarbital sodium (55 mg/kg), as the dose of GRAE increased, the number of sleeping mice and the rate of falling asleep increased. The rates of falling asleep in the LD, MD, and HD groups were all significantly higher than that in CK (Table 1, *P* < 0.01).

**Table 1:** Effect of GRAE on the effects of a subthreshold dose of pentobarbital sodium.

**Group**	**Number of sleeping mice**	**Rate (%)**
CK	1	10
LD	2	20
MD	4	40
HD	8	80

*CK, Control group; LD, Low-dose group; MD, Middle-dose group; HD, High-dose group. (n = 10)*.

**Figure 2:**
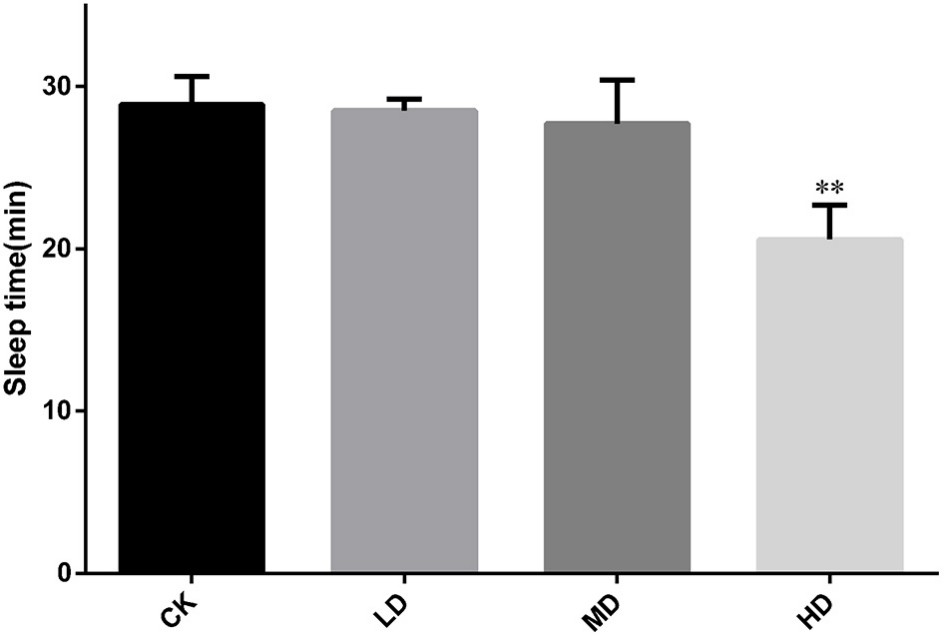
Effect of GRAE on the length of sleep latency in mice induced by barbiturate sodium in sleep improvement experiment. Each bar represents the mean ± SD (*n* = 10). *P < 0.05 vs. control group, **P < 0.01 vs. control group.

Figure 2 summarizes the sleep latency after induction by barbital sodium (280 mg/kg). As the dose of GRAE increased, the sleep latency showed a decreasing trend. The sleep latencies in the LD and MD groups were shorter than that in CK, but the differences were not significant. The sleep latency in the HD group was significantly reduced (*P* < 0.01) to 0.71 times that in CK.

### Effect of GRAE on Mouse Brain Metabolites

#### OPLS-DA

**Figure 3:**
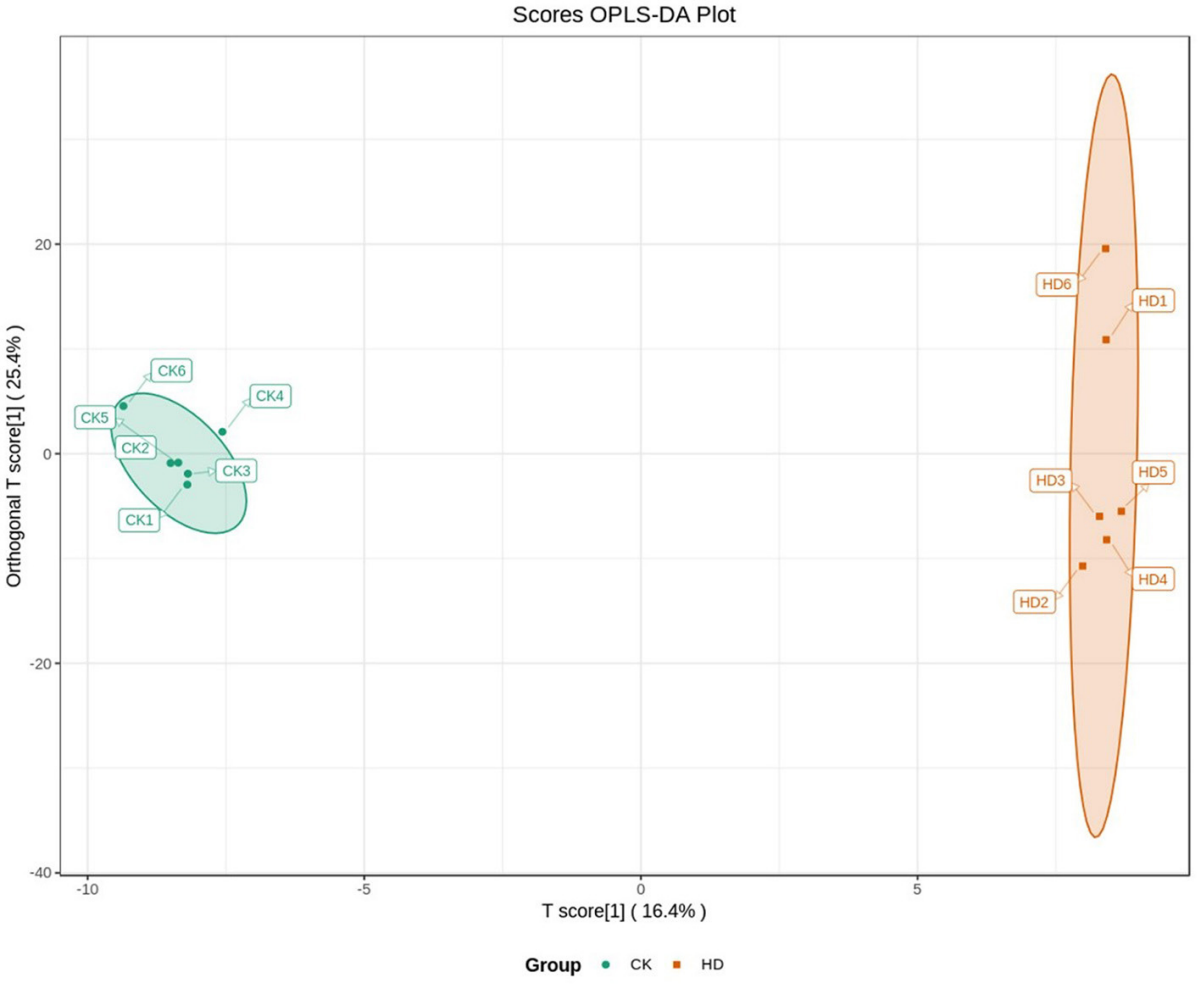
Differences brain metabolite between treatment groups in mice analyzed by OPLS-DA.

Orthogonal partial least squares discriminated analysis (OPLS-DA) can maximize the distinction between groups and is a useful approach for the identification of metabolite differences (23). The prediction parameters for the evaluation model, R^2^X, R^2^Y, and Q^2^ represent the fractions of variance explained by the X and Y matrices and the predictive ability of the model. As shown in Table 2, the Q^2^ value for the comparison between the HD and CK groups (i.e., Q^2^ > 0.5) indicates that the OPLS-DA model was effective. The Q^2^ value for the comparison between the MD and CK groups (i.e., Q^2^ < 0.5) indicates that the OPLS-DA model is invalid. The samples from the HD and CK group were completely separated and there were significant differences in the brain metabolite profiles (Figure 3).

**Table 2:** R^2^X, R^2^Y, and Q^2^ of OPLS-DA in mouse brain tissue samples from various groups.

**Index**	**R^**2**^X (cum)**	**R^**2**^Y (cum)**	**Q^**2**^ (cum)**
CK vs. MD	0.423	0.997	0.058
CK vs. HD	0.604	0.998	0.541

*Q^2^ represents the predictive ability of the model. The closer the three indicators are to 1.0, the more stable and reliable the model is. When Q^2^ > 0.5, the model is effective. When Q^2^ > 0.9 the model is excellent. CK, Control group; MD, Middle-dose group; HD, High-dose group. (n = 10)*.

The results of the sleep experiments showed that mice in the HD group had the best sleep improvement effects, compared with the CK group. OPLS-DA analysis showed that the OPLS-DA model was invalid between the MD and CK groups. Therefore, the metabolome data of the MD could not be used for subsequent analysis.

#### Targeted Metabolomic Analysis

The metabolites that were significantly different between the CK and HD groups were screened as shown in Table 3 (metabolites with VIP ≧ 1 are generally considered being significantly different). Compared with the CK group, the relative contents of 73 metabolites in the brain tissue of the HD group were significantly altered. The 73 annotated metabolites were divided into 12 categories: amino acid metabolomics, benzene and substituted derivatives, camitine, carbohydrate metabolomics, coothers enzyme factor and vitamin, indole and its derivatives, ketones, lactone, lipid fatty acids, nucleotide metabolomics, organic acid and its derivatives, oxidized lipid, phenols and its derivatives, and polyamine.

**Table 3:** Significant metabolites differences in the mouse brain between the high-dose and control groups.

**Index**	**Compounds**	**Class**	**Log_**2**_FC**	**Type**
MEDP180	B-Nicotinamide Mononucleotide	Nucleotide metabolomics	4.4381	up
MEDN335	Methylmalonic Acid	Organic Acid and Its Derivatives	4.1194	up
MEDN201	Succinic Acid	Amino Acid metabolomics	4.104	up
MEDN478	Aminomalonic Acid	Organic Acid and Its Derivatives	4.0828	up
MEDP161	Adenosine 5'-Diphosphate	Nucleotide metabolomics	3.1019	up
MEDN537	ADP-ribose	Nucleotide metabolomics	2.9998	up
MEDN065	O-Phospho-L-Serine	Amino Acid metabolomics	2.9106	up
MEDN421	Cyclic Amp	Nucleotide metabolomics	2.248	up
MEDP043	Glutathione Oxidized	Amino Acid metabolomics	2.1983	up
MEDP577	Isobutyryl carnitine	Camitine	1.9611	up
MEDN819	3-Hydroxymandelate	Organic Acid and Its Derivatives	1.8763	up
MEDN299	Adipic Acid	Organic Acid and Its Derivatives	1.7743	up
MEDN242	L-Ascorbate	CoOthersEnzyme Factor & vitamin	1.6963	up
MEDP618	2-Methylbutyroylcarnitine	Lipids_Fatty Acids	1.6788	up
MEDN797	PGJ2 [11-oxo-15S-hydroxy-prosta-5Z,9,13E-trien-1-oic acid]	Oxidized lipid	1.6104	up
MEDP002	D-Homocysteine	Amino Acid metabolomics	1.3374	up
MEDN366	Lysope 16:0	LipidsOthersPhospholipid	1.2566	up
MEDP665	Methyl isobutyl ketone	Ketones	1.1376	up
MEDN835	D-Glucuronolactone	Lactone	1.0918	up
MEDN658	Hexadecanedioic acid	Lipids_Fatty Acids	1.0698	up
MEDN659	Pyrophosphate	Organic Acid and Its Derivatives	−1	down
MEDP409	Cys-Gly	Amino Acid metabolomics	−1.001	down
MEDP367	γ-Aminobutyric Acid	Organic Acid and Its Derivatives	−1.019	down
MEDP431	Hordenine	Benzene and substituted derivatives	−1.02	down
MEDP791	4-aminophenol	Phenols and Its Derivatives	−1.02	down
MEDN228	D-Arabinose	Carbohydrate metabolomics	−1.038	down
MEDP617	2-Furoylglycine	Amino Acid metabolomics	−1.044	down
MEDP880	N-Alpha-Acetyl-L-Asparagine	Amino Acid metabolomics	−1.055	down
MEDP015	L-Citrulline	Amino Acid metabolomics	−1.074	down
MEDP859	Glycylphenylalanine	Amino Acid metabolomics	−1.081	down
MEDN615	Carbamoyl phosphate	Organic Acid and Its Derivatives	−1.084	down
MEDP395	L-Pipecolic Acid	Amino Acid metabolomics	−1.089	down
MEDN211	D-Arabitol	Carbohydrate metabolomics	−1.094	down
MEDN070	Sarcosine	Amino Acid metabolomics	−1.116	down
MEDP325	Maleic Acid	Organic Acid and Its Derivatives	−1.16	down
MEDN352	O-Phosphorylethanolamine	LipidsOthersPhospholipid	−1.16	down
MEDN499	Argininosuccinic acid	Organic Acid and Its Derivatives	−1.175	down
MEDN032	Allantoin	Organic Acid and Its Derivatives	−1.181	down
MEDP718	Methylisobutyrate	Organic Acid and Its Derivatives	−1.209	down
MEDN043	L-Carnosine	Amino Acid metabolomics	−1.231	down
MEDN170	Uridine 5′-Diphosphate	Nucleotide metabolomics	−1.271	down
MEDN686	Methyl propyl disulfide	Others	−1.28	down
MEDP296	4-Guanidinobutyric Acid	Organic Acid and Its Derivatives	−1.296	down
MEDN822	Cysteine glutathione disulfide	Amino Acid metabolomics	−1.312	down
MEDP024	L-Serine	Amino Acid metabolomics	−1.32	down
MEDP128	Diethanolamine	Polyamine	−1.323	down
MEDP060	Methionine Sulfoxide	Amino Acid metabolomics	−1.367	down
MEDN028	4-Hydroxy-L-Glutamic Acid	Amino Acid metabolomics	−1.367	down
MEDN034	Beta-Alanine	Amino Acid metabolomics	−1.37	down
MEDN707	Thiodiglycolic Acid	Organic Acid and Its Derivatives	−1.382	down
MEDN214	L-Arabitol	Carbohydrate metabolomics	−1.384	down
MEDP387	H-Homoarg-Oh	Amino Acid metabolomics	−1.401	down
MEDN327	L-Dihydroorotic Acid	Organic Acid and Its Derivatives	−1.402	down
MEDP029	Cysteamine	Polyamine	−1.411	down
MEDP014	L-Aspartic Acid	Amino Acid metabolomics	−1.435	down
MEDN007	L-Arginine	Amino Acid metabolomics	−1.437	down
MEDN480	Dl-Glyceraldehyde3-Phosphate	Organic Acid and Its Derivatives	−1.44	down
MEDN651	Succinic anhydride	Organic Acid and Its Derivatives	−1.473	down
MEDN173	Uridine 5′-Diphospho-N-Acetylgalactosamine	Nucleotide metabolomics	−1.475	down
MEDP271	3-Indolepropionic Acid	Indole and Its Derivatives	−1.501	down
MEDP504	Glucosamine	Carbohydrate metabolomics	−1.516	down
MEDP845	1-Phenylethanol	Benzene and substituted derivatives	−1.531	down
MEDN769	14(S)-HDHA [14S-hydroxy-4Z,7Z,10Z,12E,16Z,19Z-docosahexaenoic acid]	Oxidized lipid	−1.551	down
MEDP071	N-Glycyl-L-Leucine	Amino Acid metabolomics	−1.581	down
MEDP874	Tryptophan betaine	Organic Acid and Its Derivatives	−1.589	down
MEDN046	L-Glutamine	Amino Acid metabolomics	−1.713	down
MEDP049	L-Asparagine Anhydrous	Amino Acid metabolomics	−1.778	down
MEDN490	Fumaric Acid	Amino Acid metabolomics	−1.818	down
MEDP006	Glycine	Amino Acid metabolomics	−1.974	down
MEDN538	UDP-glucose	Nucleotide metabolomics	−2.021	down
MEDN463	D-Fructose 6-Phosphate-Disodium Salt	Carbohydrate metabolomics	−2.142	down
MEDP064	N-Acetylcysteine	Amino Acid metabolomics	−2.405	down
MEDN004	L-Cystine	Amino Acid metabolomics	−2.847	down

*VIP ≥ 1, the change in the metabolite is significant; FC, fold change. (n = 10)*.

Among these metabolites, 20 were upregulated in the HD group, whereas 53 were downregulated. The most significantly upregulated metabolites were B-nicotinamide mononucleotide, methylmalonic acid, succinic acid, amino malonic acid, and adenosine 5′-diphosphate. The most significantly downregulated metabolites were L-cystine, N-acetylcysteine, D-fructose 6-phosphate-disodium salt, UDP-glucose, and glycine.

#### Transcriptomics Analysis of Mice Brain Tissue

**Figure 4:**
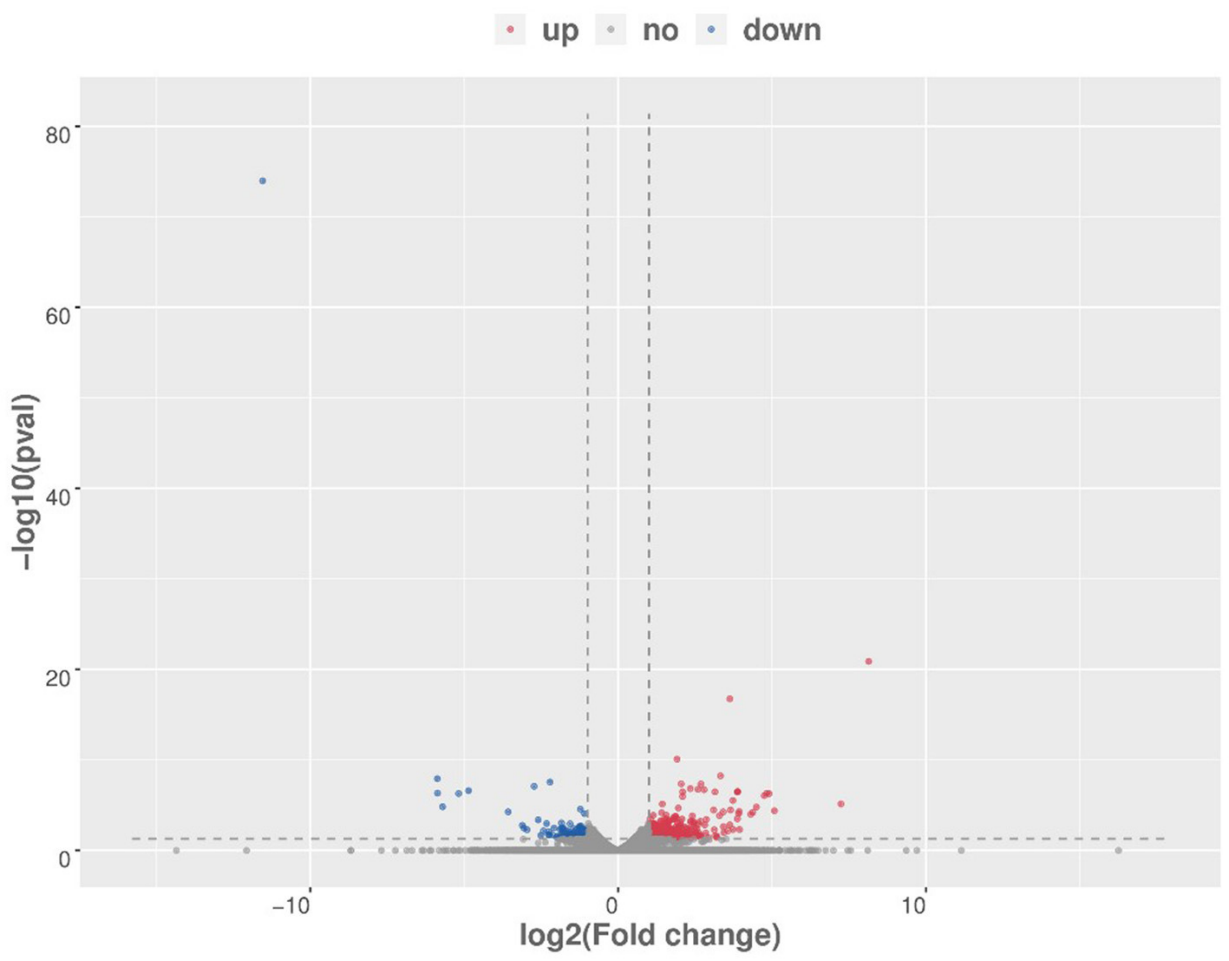
Volcano map of differential genes (HD vs. CK). Red represents upregulated significantly differentially expressed genes, blue represents downregulated significantly differentially expressed genes, and gray dots represent non-significant differentially expressed genes. HD, High-dose group; CK, Control group.

**Figure 5:**
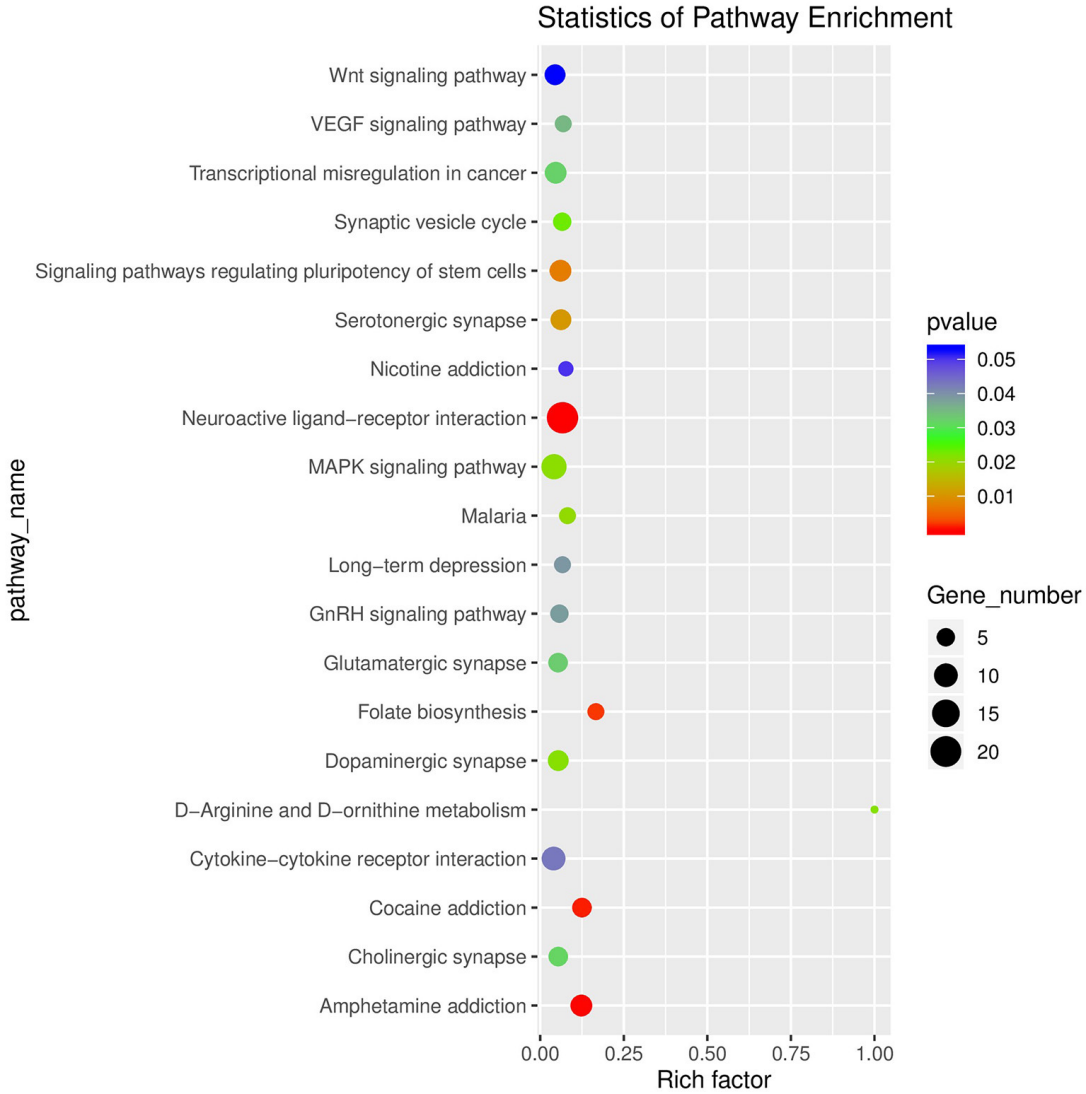
Enrichment diagram of significantly different genes based on a KEGG analysis (top 20). The rich factor referred to the ratio of the number of deferential expressed genes to the total number of genes enriched in a specific category. The size of circles roughly represented the count of deferential expressed genes. The color saturation from blue to red indicated *P*-value (Student-Newman-Keuls test, *n* = 3).

After raw data filtering, sequencing error rate checking, and GC content distribution checking, the clean reads data used for subsequent analysis were obtained. The Q20% (sequencing error rate < 0.01) was 99.6% and the Q30% (sequencing error rate < 0.001) was 97.63–98.19%. Transcriptome data was then used for further analysis. The brain tissues of mice in the HD and CK groups were screened and a total of 500 significantly different genes were obtained, of which 373 genes were significantly (*P* < 0.05) upregulated and 127 genes were significantly (*P* < 0.05) downregulated (Figure 4). These differential genes were enriched in 99 pathways. KEGG enrichment results showed the genes were mainly enriched in neuroactive ligand-receptor interaction, amphetamine addiction, and cocaine addiction (Figure 5). The raw sequencing data of transcriptome of mouse brain were submitted to Sequence Read Archive (SRA) database (with SRA number: SRR17023841- SRR17023846).

#### Conjoint Analysis of Transcriptome and Metabolome

**Figure 6:**
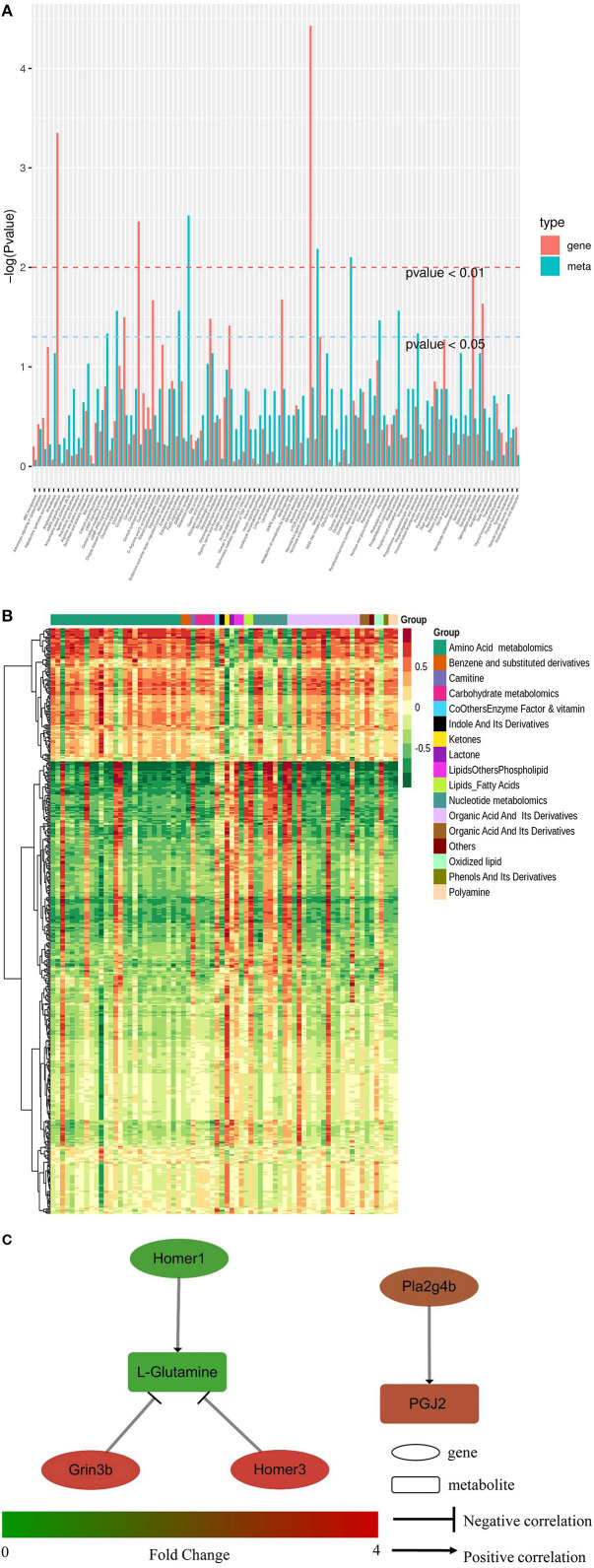
Conjoint analysis of transcriptome and metabolome in mice brain tissues of CK and HD group. **(A)** KEGG pathway enrichment of differential genes and metabolites. **(B)** Heatmap of correlation coefficient matrix between differential metabolites and differential genes with the Pearson correlation coefficient above 0.8. Red and green indicated the positive and negative correlation, respectively. The color labels represent Pearson correlation coefficient values. **(C)** Regulating network between the genes and metabolites in sleep-related pathways. Differences in expression levels between the HD and CK groups was indicated by different colors, with green indicating down-regulation and red indicating up-regulation.

Conjoint analysis of the transcriptome and metabolome was used to determine correlations between genes and metabolites. Enrichment analysis was performed for differential genes and metabolites between the HD and CK groups. As shown in Figure 6A, the differential metabolites and genes were both enriched in 102 KEGG pathways, including eight pathways potentially related to sleep, namely MAPK signaling pathway, cAMP signaling path-way, neuroactive ligand-receptor interaction, circadian rhythm, circadian entrainment, glutamatergic synapse, serotonergic synapse, and GABAergic synapse. A total of 10 differential metabolites and 42 differential genes were enriched in eight sleep-related pathways.

The Pearson correlation coefficients (PCC) between genes and metabolites were calculated using the cor program in R. The differential genes and metabolites with PCC higher than 0.8 were selected and a clustered heatmap (Figure 6B) was drawn. The clustered heatmap showed that the differential metabolites associated with differential genes could be classified into 17 categories, among which amino acid metabolism was the largest category.

The regulation network between the screened differential genes and metabolites was analyzed to further understand the regulatory relationships between the sleep-related differential genes and metabolites. As shown in Figure 6C, L-glutamine was negatively regulated by *Homer3* and *Grin3b*, but *Homer1* positively regulated it. PGJ2 (11-oxo-15S-hydroxy-prosta-5Z, 9, 13E-trien-1-oic acid) was positively regulated by Phospholipase A2 Group IVB gene (*Pla2g4b*).

## Discussion

This study showed that 30 days of GRAE (1,000 mg/kg.bw) administration can prolong pentobarbital sodium-induced sleep in mice, increase the sleeping rate in mice treated with a subthreshold pentobarbital sodium dose, and shorten the sleep latency. These results indicate that *G. resinaceum* prolongs sleep time. After daily administration *via* gavage, the mice did not sleep. Therefore, GRAE does not directly induce sleep in mice.

To explore the mechanism by which GRAE prolongs sleep in mice, a comprehensive targeted metabolic analysis was performed using a combination of UPLC-MS/MS and statistical analyses to identify metabolite differences between the treatment groups and the control group (21, 24). OPLS-DA revealed significant differences in mice brain metabolites between the CK and HD groups. Identification analysis of differential metabolites in mice brain tissues showed GRAE has a substantial impact on amino acid metabolism. Amino acids and their derivatives play important roles in neurotransmitter activity and their alterations are expected to affect sleep (25). In this study, the sleep time of mice in the HD group was significantly longer than in the CK group, and the contents of L-glutamine in brain tissue of mice in HD group were reduced. L-glutamate is an excitatory amino acid, glutamate and glutamine can be interconverted and the glutamate-glutamine cycle is crucial for the proper maintenance of synaptic activity in brain tissue (26). The results of previous studies showed that increased glutamine and glutamate levels can reduce non-rapid eye movement sleep, which is consistent with the results of this study (27).

Conjoint analysis of transcriptome and metabolome revealed that the most remarkably enriched pathway for differential genes enrichment is the neuroactive ligand-receptor interaction pathway, which is enriched to 19 genes and five metabolites. Bioinformatics analysis showed that upregulation of neuroactive ligand-receptor interactions improved sleep (28). The most noticeably enriched pathway for differential metabolites enrichment is the GABAergic synapse pathway, which is upregulated and enriched to four metabolites and two genes. The GABAergic pathway may adaptively tune the neural property of dorsal fan-shaped body neurons to temperature shifts and reorganize sleep architecture (29), which is consistent with the results in this study that increased mice sleep duration upregulated the GABAergic pathway. Studies have also shown that circadian rhythm, circadian entrainment, cAMP signaling pathway, glutamatergic synapse, serotonergic synapse and MAPK signaling pathway were associated with sleep. Circadian rhythm and circadian entrainment control light/dark cycle, and light has been shown to modulate autonomic and neuroendocrine responses as well as regulating sleep such as attention and arousal (30). Sleep deprivation impaired 3′, 5′-cyclic AMP (cAMP) dependent forms of synaptic plasticity in the mouse hippocampus, reduced cAMP signaling, and drugs that enhance cAMP signaling may counteract the effects of sleep deprivation (31). Glutamate is an excitatory neurotransmitter that affects mood, and glutamatergic neurotransmission is associated with antidepressant-like effects (32). Glutamatergic synapse influences glutamatergic neurotransmission, which consequently affects sleep through mood. Serotonin inhibits rapid eye movement sleep through an action on cholinergic neurons in the mesopontine tegmentum (33). Although no evidence for a directly effect of MAPK signaling pathway on sleep, but some studies have found obstructive sleep apnea (OSA) activated mitogen-activated protein kinase (MAPK) family members.

KEGG analysis of differentially expressed genes and metabolites showed that 10 differential metabolites and 42 differential genes were enriched in eight sleep-related pathways, of which two differential metabolites showed a strong correlation with four differential genes [Pearson correlation coefficient (PCC), |PCC| > 0.8]. As the regulating network between the genes and metabolites showed that two homer scaffolding protein genes, *Homer1* and *Homer3*, are correlated with L-glutamine in glutamatergic synapse pathway. This agrees with previous findings that Homer proteins form metabotropic glutamate receptors with several actors at critical key points of signaling pathways, *Homer1* and *Homer3* regulates the trafficking and surface expression of Type I metabotropic glutamate (mGlu1) receptors, and Homer proteins form metabotropic glutamate receptors with several actors at critical key points of signaling pathways (34, 35). In the glutamatergic synapse pathway, GRAE increased the expression of *Grin3b* and decreased the expression of L-glutamine. NR3B, encoded by the *Grin3b* gene, can protect motoneurons against glutamate-mediated excitotoxicity (36). Blocking NMDA receptors (NR3B) *in vivo* increases glutamine synthetase activity and glutamine content in brain, and then affect the content of glutamate through the glutamate-glutamine cycle (37). This result indicated that GRAE may affect the accumulation of L-glutamine in mice brain tissue by regulating the expression of *Grin3b*. This study showed that *Homer1, Homer3*, and *Grin3b* were related to L-glutamine, which indicates that they may play a role in regulating L-glutamine accumulation. This study is the first to find that GEAE improves sleep in mice from both the metabolome and transcriptome, and this relationship correlates with L-glutamine levels, raising the conclusion that *Grin3b* is associated with L-glutamine synthesis. Of course this study has its limitation, such as it cannot probe deeply how the three genes, *Homer1, Homer3*, and *Grin3b*, regulate L-glutamine synthesis in mouse brain tissue then regulate the sleep of mice, using knockout technology and neurochemical approaches in future studies. The study also showed that PGJ2 was positively regulated by *Pla2g4b*, but the mechanism of regulation has not been reported.

Rodent models have widely been used for sleep experiments due to the high similarity of brain circuitry and electrophysiological rhythms between rodents and human. In particular, both rodents and infants exhibit a short sleep-wake cycle (38). Experimental mice share the same genetic background, and can be manipulated easily, reproducibly and reliably. As a result, mice are useful animal models to reproduce an important aspect of human sleep behavior (rapid eye movement sleep). Conversely, rodents are different from humans in that rodent sleep is polyphasic, with multiple sleep-wake cycles per day and each cycle lasting from a few minutes to a several hours (39). Effects of the difference to results remain unknown, and require follow-up studies.

## Conclusions

This study investigated the effects of different concentrations of GRAE on the sleep time of mice. Our study showed that GRAE (1,000 mg/kg.bw) could prolong the sleep time of mice. This effect may be related to amino acids and their derivatives in mice brain tissue, especially the accumulation of L-glutamine and PGJ2. The conjoint analysis of the transcriptome and metabolome showed that *Homer1, Homer3*, and *Grin3b* were related to the accumulation of L-glutamine. Therefore, these genes may play a role in regulating the biosynthesis of L-glutamine in mice brain tissue. The current research provides new insights into the function of GRAE in prolonging the sleep time of mice.

## Data Availability Statement

The datasets presented in this study can be found in online repositories. This data can be found here: https://www.ncbi.nlm.nih.gov/sra?linkname=bioproject_sra_all&from_uid=781743 [accession: SRR17023841 – SRR17023846].

## Ethics Statement

The animal study was reviewed and approved by the Animal Care and Use Committee of the College of Food Science, Fujian University of Agriculture and Forestry (Protocol code number FS-2019-0055, approved on October 10, 2019).

## Author Contributions

TC: writing review and editing, data curation, investigation, and validation. FZ and RW: writing—original draft, data curation, and investigation. JC, QZ, and YH: data curation and investigation. BX: funding acquisition and conceptualization. YJ: funding acquisition, conceptualization, methodology, formal analysis, and project administration. BC: funding acquisition, conceptualization, methodology, formal analysis, writing—original draft, project administration, and writing—review and editing. All authors contributed to the article and approved the submitted version.

## Funding

This research was supported by the National Key Research and Development Program of China (2019YFC1710500), National Natural Science Foundation of China (31801920), National Modern Agriculture Technology System of Edible Fungi Industry (KMD16001A), Natural Science Foundation of Fujian Province (2020J01557), Education and Scientific Research Projects of Young and Middle-aged Teachers in Fujian Province (JT180115), Fujian Agriculture and Forestry University Science and Technology Innovation Fund Project (CXZX2017409 and CXZX2017410) and Natural Science Foundation for Distinguished Young Scholar of Fujian Agriculture and Forestry University of China (xjq202113, BC).

## Conflict of Interest

The authors declare that the research was conducted in the absence of any commercial or financial relationships that could be construed as a potential conflict of interest.

## Publisher's Note

All claims expressed in this article are solely those of the authors and do not necessarily represent those of their affiliated organizations, or those of the publisher, the editors and the reviewers. Any product that may be evaluated in this article, or claim that may be made by its manufacturer, is not guaranteed or endorsed by the publisher.
